# Clinical and Imaging Resolution of Neonatal Hemochromatosis following Treatment

**DOI:** 10.1155/2014/650916

**Published:** 2014-06-24

**Authors:** Ayelet Machtei, Gil Klinger, Rivka Shapiro, Osnat Konen, Lea Sirota

**Affiliations:** ^1^Neonatal Intensive Care Unit, Schneider Children's Medical Center of Israel, 14 Kaplan Street, 49202 Petah Tikva, Israel; ^2^The Sackler School of Medicine, Tel Aviv University, Tel Aviv, Israel; ^3^Institute of Gastroenterology, Nutrition and Liver Diseases, Schneider Children's Medical Center of Israel, Petah Tikva, Israel; ^4^Radiology Department, Schneider Children's Medical Center of Israel, Petah Tikva, Israel

## Abstract

Neonatal hemochromatosis (NH) is an acute liver disease associated with both hepatic and extrahepatic iron deposition and is a leading cause of neonatal liver transplantation. The concept that NH is an alloimmune disease has led to the emergence of a new treatment approach utilizing exchange transfusion and intravenous immunoglobulin therapy. We present a two-day old neonate with progressive liver dysfunction who was diagnosed with NH. Magnetic resonance imaging confirmed tissue iron overload. Treatment with intravenous immunoglobulins and exchange transfusion led to rapid improvement in liver function. Follow-up physical examination at the age of 8 months showed normal development and near normal liver function. A repeat abdominal magnetic resonance scan at 8 months showed no signs of iron deposition in the liver, pancreas, or adrenal glands. The present report provides further support for the use of exchange transfusion and immunoglobulin therapy in NH and is the first to document resolution of typical iron deposition by magnetic resonance imaging.

## 1. Introduction 

Neonatal hemochromatosis (NH) is a rapidly progressive disease presenting within a few days after birth with fulminant hepatic failure and ensuing multiorgan failure. NH is also known as neonatal iron storage disease or congenital alloimmune hepatitis. For many years the only curative treatment for NH was liver transplantation with survival rates of 50% [1]. Treatment with antioxidants and chelation therapy may improve symptoms but is associated with severe side effects [2]. In recent years the realization that NH is probably an alloimmune disease [3, 4] has led to the emergence of a new treatment approach utilizing exchange transfusion (ET) and intravenous immunoglobulins (IVIG). This has resulted in an improved survival rate and in a dramatic decrease in the need for liver transplantation. The understanding of the pathophysiology of the disease has also led to antenatal treatment with IVIG from 16 weeks' gestation and has been shown to prevent the development of NH in subsequent pregnancies [4]. In the present report we present a full-term newborn with liver dysfunction and multiorgan failure, diagnosed with NH that recovered fully following treatment with IVIG and ET.

## 2. Case Report

A female neonate was born at 39 weeks of gestation following an uneventful pregnancy. The infant was delivered by vacuum extraction due to a profound deceleration. Apgar scores at birth were 9 and 10 at 1 and 5 minutes, respectively. The infant weighed 2.724 kg (10th percentile) and the initial physical examination was normal. The patient was the first born (and first gestation) to healthy nonconsanguineous Ashkenazi Jews. At the age of two days the mother reported a decreased appetite and subsequently the infant's condition deteriorated rapidly. Physical examination showed pallor, hypothermia (35.9°C), and bradycardia of 70 beats per minute (bpm). The initial laboratory evaluation showed hypoglycemia of 13 mg/dL (normal lower limit of 40 mg/dL). The patient was treated with intravenous boluses of 10% glucose and normal saline and was transferred to the neonatal intensive care unit (NICU). Upon admission to the NICU the infant's vital signs were as follows: temperature of 35.8°C, heart rate of 116 bpm, breath rate of 51 per minute, and blood pressure of 61/33 mm/Hg. Physical examination showed a lethargic infant with glucose level of 19 mg/dL; thus, a second bolus of 10% glucose was administered. During the hypoglycemic episode a critical blood sample was taken (insulin, cortisol, growth hormone, thyroid function, and lactate) and a full sepsis workup (complete blood count, C-reactive protein level, blood culture, and cerebrospinal fluid analysis and culture) was performed. Additional tests drawn included blood chemistry, a coagulation panel, and tests for possible metabolic abnormalities. Treatment with ampicillin, gentamycin, and acyclovir was initiated. Initial blood tests showed leukocytosis, elevated liver enzymes and creatinine, and evidence of coagulopathy (Table 1). The remaining endocrinological parameters were within normal limits. During the next few days the patient's condition deteriorated. Although she remained normoglycemic, her liver function worsened progressively; coagulation tests showed disseminated intravascular coagulation (DIC) despite treatment with platelets, fresh frozen plasma, and vitamin k. A workup for possible hepatitis causing pathogens turned out negative. The metabolic evaluation was negative (including blood carnitine, acyl carnitine, amino acids, very long chain fatty acids, galactosemia, pyruvate dehydrogenase, E3 deficiency, and congenital disorder of glycosylation). Alpha 1 antitrypsin level was normal, alpha-fetoprotein was high (143,621 ng/mL) compared to normal values [5], iron was 155 *μ*g/dL (normal 40–145), and ferritin was extremely elevated (24,256 ng/mL) compared to standard range (10–291). An abdominal ultrasound showed normal hepatic and bile ducts and a moderate degree of ascites. Because of suspected convulsions the patient underwent a head ultrasound which showed mild cerebral edema and an electroencephalogram that was normal.

**Figure 1:**
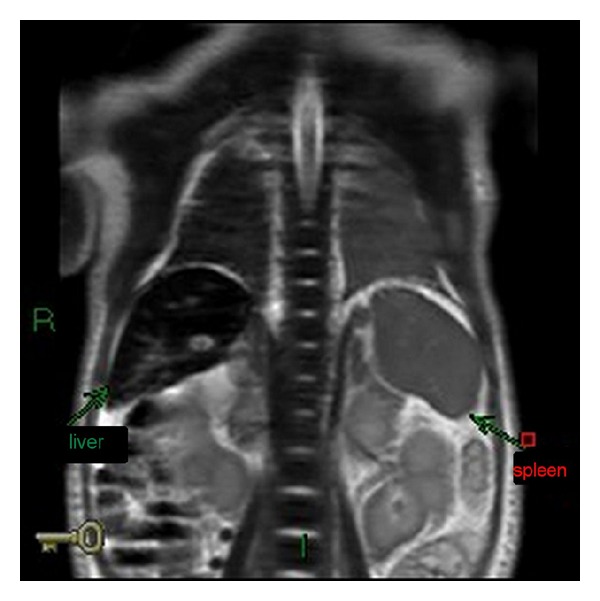
Abdominal MRI, coronal view, on day five of life showing low signal intensity of the liver parenchyma with preservation of the signal intensity of splenic parenchyma (T2WI protocol). Calculations of T2∗ showed rapid decay consistent with iron overload.

Based on the clinical and laboratory findings we suspected NH to be the cause of the infant's condition and performed an abdominal magnetic resonance imaging (MRI) scan and a buccal biopsy. The MRI showed a clear shortening of the T2 signal to 3.5–5.5 ms (normal 25–30 ms) from the liver and pancreas which is characteristic of tissue iron overload (Figure 1). The buccal biopsy was negative for iron staining, as was a repeat biopsy. Treatment was initiated on day 8 with double volume exchange transfusion and IVIG 2 gr/kg as well as with vitamin E 25 IU three times per day. Following treatment, the patient showed rapid clinical and laboratory improvement (Table 1): platelets count (76,000 to 142,000), INR (2.83 to 1.99), ammonia (502 to 149), and creatinine (1.33 to 0.16). The patient was discharged home on day 22 of life.

**Figure 2:**
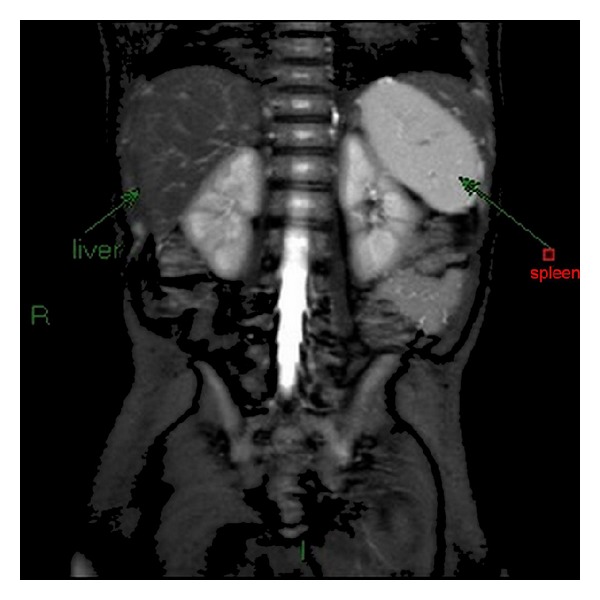
Follow-up abdominal MRI, coronal view, at 8 months showing normal intensity of liver parenchyma on T2WI with fat saturation protocol.

The infant has continued the follow-up by our Gastroenterology outpatients service. At the age of 8 months the infant was healthy, developing normally, and liver function tests were approaching normal values. A repeat abdominal MRI at the age of 8 months was normal and showed no signs of iron deposition in the liver, pancreas, or adrenal glands as compared to the spleen (Figure 2).

## 3. Discussion

NH is a rare condition affecting the fetus and newborn that presents during the neonatal period with acute liver failure and ensuing multiorgan failure. Untreated NH is uniformly fatal. No single gene has been recognized as being responsible for the disease [3]. In families with one affected child there is a 70–80% chance of recurrence in subsequent pregnancies [4], a recurrence rate that is not compatible with either an autosomal recessive or dominant inheritance pattern. Mitochondrial inheritance has been suggested but has not been confirmed [1, 2, 6].

Knisely at al. [7] and Whitington and Malladi [8] suggested that the pattern of recurrence in NH resembles that of gestational alloimmune diseases in which the maternal immune system develops antibodies of the immunoglobulin G class which then cross the placenta and attack the fetal liver (in the current and subsequent pregnancies). In NH this process causes synthetic dysfunction of the different proteins involved in iron homeostasis which results in siderosis.

Patients with NH may present during pregnancy with intrauterine growth retardation, oligohydramnios (or, at times polyhydramnion), and placental edema and are often born prematurely. Within hours or days they develop acute liver failure. Frequently the infants are at first misdiagnosed with overwhelming sepsis. Laboratory abnormalities include hypoglycemia, hypoalbuminemia, and marked coagulopathy. Jaundice with elevated direct and indirect hyperbilirubinemia develops within days. Serum aminotransaminases are disproportionately low for the degree of hepatic injury, while alpha-fetoprotein levels are very high. Characteristic iron studies show low levels of transferrin that is unusually highly saturated and extremely elevated ferritin levels (150–450 K/micl; [9]). The diagnosis of NH can be difficult to establish because other hepatic disorders may cause liver siderosis or high ferritin levels. The diagnosis is made by exclusion of other causes of neonatal liver failure and by demonstrating extrahepatic siderosis with reticuloendothelial sparing (as seen in hereditary hemochromatosis). Extrahepatic siderosis may be demonstrated by biopsy of the minor salivary glands that are positive in about two thirds of infants with proven NH [9] or by MRI, which demonstrates abnormal iron distribution in about 90% of proven cases. Ferric ions cause shortening of the T1 and, more impressively, of the T2 relaxation times. In NH, tissues that contain iron, such as the liver and pancreas, have a lower intensity signal than that of the normal spleen; although it should be noted that a physiological increase in iron content of the liver (alone) exists during the third trimester and neonatal period. An MRI can therefore be used for third trimester prenatal diagnosis.

Liver biopsy demonstrates nodular cirrhosis, pronounced fibrosis, and typically significant siderosis. Notably there are no signs of necrosis.

Until recently liver transplantation was considered the only curative treatment for NH with survival rates as high as 50%. Use of an ET for treatment of NH was first reported in 2005 by Rodrigues et al. [1]. In their report of 19 neonates, 17 with NH were treated with ET as a supportive measure. All these treated patients either died or needed subsequent liver transplantation. However, one should note that in this report, ET was given as a last measure to severely compromised patients. Whitington and Hibbard [4] described in 2004 a preventive prenatal treatment for pregnant women whose last pregnancy resulted in an infant or fetus with NH (based on the alloimmune theory). Treatment consisted of IVIG 1 g/kg weekly starting at 18 weeks of gestation (at which point maternal IgG antibodies are actively transported across the placenta). They reported 16 pregnancies that progressed uneventfully and resulted in all infants surviving. High dose immunoglobulin therapy during pregnancy for recurrent NH has since become common practice. In 2009 Rand et al. [10] published the largest series to date of neonates diagnosed with NH and treated by ET at twice the calculated blood volume and/or IVIG 1 g/kg. The ferritin levels were as high as 5509 ng/mL. Twelve subjects (75%) had a good outcome following treatment (poor outcome defined as death or receipt of a liver transplant). Since the initial report, additional reports by Lopriore et al. [11] and Babor et al. [12] have confirmed the effectiveness of ET and IVIG for NH.

The present report includes a newborn diagnosed with NH that presented with untypical severe liver failure. As this infant was the firstborn in her family the patient was only diagnosed after birth, thus adversely affecting the severity of the NH. Our report confirms that even when NH is severe, combined treatment with IVIG and exchange transfusion may achieve complete symptom resolution. The reported infant is the first to demonstrate resolution of iron deposition confirmed by follow-up MRI scan. We add our experience to the accumulating data supporting combined neonatal treatment with IVIG and exchange transfusion.

## Figures and Tables

**Table 1:** Laboratory values before and after treatment.

	Reference values	Peak value	After treatment
International normalized ratio	0.8–1.20 INR	2.83	1.99
Platelets	150–450 K/micl	76	142
Bilirubin total	0.3–1.2 mg/dL	11	8.4
Bilirubin direct	0.3 mg/dL	3.3	3.8
Aspartate transaminase	25.0–75.0 U/L	727	181
Alanine transaminase	13.0–45.0 U/L	196	114
Albumin	2.8–4.4 g/dL	2.2	2.9
Phosphorus	4.0–6.5 mg/dL	4.3	5.8
C-reactive protein	0.0–0.50 mg/dL	1.552	0.4
Iron	40–145 *µ*g/dL	155	
Ferritin	10.0–291.0 ng/mL	24256	3030
Alpha-fetoprotein	Mean 41,687 ng/mL	143621	94709
Ammonia	0–228 mcg/dL	502	149
Urea	8–26 mg/dL	70	11
Creatinine	0.26–1.01 mg/dL	1.33	0.16

## Abbreviations

NH: Neonatal hemochromatosis
IVIG: Intravenous immunoglobulin
ET: Exchange transfusion
NICU: Neonatal intensive care unit
MRI: Magnetic resonance imaging.
